# Distribution of phenotypes of coronal plane alignment of the knees and functional phenotypes in the healthy young Iranian population: A cross‐sectional study

**DOI:** 10.1002/jeo2.70229

**Published:** 2025-04-16

**Authors:** Mohammadreza Razzaghof, Mohammad Soleimani, Mohammad Poursalehian, Mohammad Ayati Firoozabadi, Seyed Amirsadegh Mortazavi, Seyed Mohammad Javad Mortazavi

**Affiliations:** ^1^ Department of Orthopedic Surgery, Imam Khomeini Hospital Complex Tehran University of Medical Sciences Tehran Iran; ^2^ Joint Reconstruction Research Center Tehran University of Medical Sciences Tehran Iran

**Keywords:** alignment, anatomical alignment, CPAK, FMA, HKA, joint line obliquity, knee, mechanical alignment, MPTA, phenotype, TMA, valgus, varus

## Abstract

**Purpose:**

The functional phenotypes and the coronal plane alignment knee (CPAK) classification have recently been introduced. This study aimed to describe the phenotypes of normal knees in the healthy young Iranian population based on these systems.

**Methods:**

This cross‐sectional study included 402 knees in 201 healthy young adults. Phenotypes were reported using hip–knee–ankle (HKA) angle, femoral mechanical angle (FMA) and tibial mechanical angle (TMA) for functional phenotypes and joint line obliquity (JLO) and arithmetic HKA (aHKA) for CPAK classification. Also, a distinct combination of knee alignment parameters (HKA, FMA and TMA) that reflects the functional alignment characteristics of the knee, as described by Hirschmann, was used. Statistical analysis was performed using the Student's *t* test.

**Results:**

The mean HKA, FMA and TMA were 176.03 ± 3.01°, 92.01 ± 2.77° and 86.52 ± 2.73°, respectively. All angles of female participants were significantly more varied compared to men (*p *< 0.05). The most common phenotypes were VAR(Varus)_HKA_3°, NEU(Neutral)_TMA_0° and NEU_FMA_0°. The distribution of the 10 most common functional phenotypes was not significantly different between men and women (*p* = 0.201). The most common CPAK types were type I (33.6%), II (26.6%) and III (16.7%), and types VII, VIII and IX were rarely seen (2.0% overall). The distribution of CPAK phenotypes significantly differed between men and women (*p* = 0.015), and CPAK type III was more frequently seen in women. Clinically, the predominance of varus phenotypes and the sex‐specific differences observed underscore the importance of tailoring surgical alignment strategies to individual anatomy.

**Conclusion:**

In conclusion, our study reveals that knee alignment in the healthy Iranian population tends to exhibit neutral or mild varus characteristics, with significant differences between men and women. These findings suggest that ethnicity may play a role in the natural alignment of the knee, which has important implications for surgical planning and outcomes.

**Level of Evidence:**

Level III.

**Abbreviations**
aHKA: arithmetic hip‐knee‐ankle
AP: anteroposterior
CPAK: coronal plane alignment of the knee
FMA: femoral mechanical angle
HKA: hip–knee–ankle
JLO: joint line obliquity
KA: kinematic alignment
LDFA: lateral distal femoral angle
MA: mechanical alignment
MPTA: medial proximal tibial angle
SD: standard deviation
TKA: total knee arthroplasty
TMA: tibial mechanical angle

## INTRODUCTION

Mechanical alignment (MA) in total knee arthroplasty (TKA) provides durable implant survivorship but overlooks the variability in knee anatomy, potentially causing soft tissue imbalance and non‐native limb alignment [1, 25]. Kinematic alignment (KA) aims to restore native, pre‐arthritic alignment, with improved balance and outcomes [33], yet accurately estimating an individual's pre‐arthritic alignment remains challenging [10, 17].

Traditional classifications (neutral, varus and valgus) offer only a broad framework [29]. More nuanced systems, such as coronal plane alignment knee (CPAK) by MacDessi et al. [20], and the classification by Hirschmann et al. [11, 13], integrate joint line obliquity (JLO), arithmetic HKA angles, FMA and TMA to capture diverse phenotypes, improving individualized surgical planning [23]. CPAK in particular provides a standardized framework that can potentially reduce complications and improve outcomes, but has been used mostly in osteoarthritic or TKA populations, leaving a gap in healthy cohorts. Insights into normal alignment are crucial for refining KA techniques and replicating native knee anatomy, thus enhancing implant positioning, soft tissue balance and long‐term outcomes.

Although knee phenotypes have been studied extensively in OA and arthroplasty patients [21, 26, 31, 32], little research examines healthy young populations [16, 20]. Our study addresses this gap by evaluating knee phenotypes in healthy young Iranians—an underrepresented demographic—to establish normative data, highlight potential regional variations and refine classification systems like CPAK. This information may guide personalized surgical strategies, improve clinical outcomes, and bolster our understanding of knee alignment across diverse populations.

## METHODS AND MATERIALS

### Study design and setting

This cross‐sectional study was conducted at Imam Khomeini Hospital Complex (IKHC), Tehran, Iran, from April 2020 to April 2023. The study protocol received approval from the institutional review board and Ethics Committee of Tehran University of Medical Sciences (IR.TUMS.IKHC.REC.1403.002). Written informed consent was obtained from all participants prior to their inclusion in the study.

### Eligibility criteria

Inclusion criteria were participants aged 15–50 years who presented to the general orthopaedics clinic without any complaints related to the lower extremities and who volunteered to participate in our study.

The exclusion criteria were history of knee ligament injury, previous knee surgery, metabolic bone disease, rheumatologic diseases, corticosteroid use, knee trauma, developmental dysplasia of the hip and patients with a history of trauma to the lower extremities, including knee trauma as well as non‐knee trauma such as proximal femoral or distal tibial fractures. To exclude early‐stage knee osteoarthritis, all participants underwent a clinical examination to assess pain, range of motion and ligament stability. Additionally, AP and lateral knee radiographs were evaluated to rule out joint space narrowing, osteophyte formation, or any signs of cartilage degeneration indicative of early osteoarthritis. Participants with radiographic or clinical findings suggestive of early osteoarthritis were excluded. For participants with one knee meeting the inclusion criteria, both knees were excluded. A total of 402 knees from 201 patients met the eligibility criteria and were enroled in the study.

The term ‘healthy population’ refers to participants who met all inclusion criteria and none of the exclusion criteria [24]. This ensured that the cohort represented a group with normal, non‐pathological knee alignment. For the assessment of osteoarthritis, we used Kellgren–Lawrence criteria as the definition of osteoarthritis.

### Radiologic protocol

Standardized radiographic imaging was performed for all participants to ensure consistency and reliability in assessing coronal plane alignment. The protocol included three essential views: anteroposterior (AP), lateral and three‐joint (full‐length alignment) views.

For the AP view, participants were positioned standing with their weight evenly distributed on both legs to simulate a natural weight‐bearing posture. The x‐ray beam was centred at the knee joint and directed perpendicular to the cassette. A standardized source‐to‐film distance of 100 cm was maintained to ensure uniform image scaling.

The lateral view was performed with participants standing and knee flexing slightly (approximately 30°). The x‐ray beam was directed laterally, centred on the knee joint, to provide a clear view of the joint space and surrounding structures.

The three‐joint alignment view included the hip, knee and ankle joints in a single exposure to evaluate the mechanical axis. Participants were positioned upright with both legs fully extended and the patella facing forward. A radiolucent ruler was placed alongside the limb to ensure accurate scaling and measurements. The x‐ray source was positioned at a fixed distance of 100 cm from the cassette.

All radiographs were obtained using a digital radiography system with a resolution of at least 300 dpi, ensuring high‐quality imaging. The images were reviewed for clarity and proper visualization of joint margins and alignment landmarks. Key measurements, such as the mechanical axis deviation (MAD) and coronal plane alignment, were conducted using MediCAD v3.5 software to enhance accuracy and reduce measurement errors.

### Radiologic assessment

All participants underwent a long AP standing radiograph of the lower limbs (alignment view) to assess alignment angles. Functional knee phenotypes were classified according to the system developed by Hirschmann et al. [13]. This system evaluates the hip–knee–ankle (HKA) angle, the femoral mechanical angle (FMA) and the tibial mechanical angle (TMA).

*HKA angle*: The medial angle between the mechanical axes of the femur and tibia.

*FMA*: The medial angle between the tangent to the distal femoral condyles and the femoral mechanical axis.

*TMA*: The medial angle between the tangent to the proximal tibial joint surface and the tibial mechanical axis.

It is important to note that HKA, FMA and TMA represent medial angles to avoid confusion with measurements such as the mechanical lateral distal femoral angle (mLDFA).

Phenotypes were identified based on these angles, with specific mean values and a range of ±1.5° from the mean. Mean values were set in 3° increments from the overall rounded mean (HKA: 180°; FMA: 93° and TMA: 87°). Phenotypes were classified by the direction of alignment (neutral, varus and valgus) and the measured angle [14].

Additionally, the CPAK classification was utilized to describe knee phenotypes. This system incorporates JLO and aHKA: The medial proximal tibial angle (MPTA) and LDFA were measured based on the mechanical axis to calculate JLO and aHKA.

*JLO*: Calculated by summing the MPTA and LDFA. A JLO of 180 ± 3° was considered neutral; values > 183° or <177° indicated an apex proximal or apex distal joint line, respectively.

*aHKA*: Calculated as MPTA minus LDFA. A value of 0 ± 2° was considered neutral; values < −2° and >2° indicated varus and valgus alignment, respectively.

To ensure the reliability of the measurements, three orthopaedic surgeons independently evaluated the HKA, TMA and FMA angle in 25 patients, and one of them repeated the evaluations one week later. Also, The definitions of neutral in the Hirschmann and CPAK classifications differ, so caution is required. In the Hirschmann classification, FMA is stated as 93° and TMA is 87°, with HKA defined as 0° being neutral. In contrast, the CPAK classification defines mLDFA as 90°, MPTA as 90° and aHKA as 0° as neutral.

### Statistical analysis

Statistical analyses were performed using Statistical Package for the Social Sciences version 25.0, and scatter plots were created using Prism 10. The post hoc power analysis was performed using G*Power 3.1. Categorical data were reported as numbers and percentages, while quantitative variables were expressed as means and standard deviations (SDs). Kolmogorov–Smirnov test was used to evaluate the normality of data. Continuous variables were compared between groups using independent *t* tests or the Mann–Whitney *U* test. The chi‐square test was used to compare the distribution of knee phenotypes between men and women. We used intraclass and interclass correlation coefficients (ICCs) to evaluate the reliability of measurements. A *p* value of less than 0.05 was considered statistically significant.

## RESULTS

### Demographics of the participants

A post hoc power analysis showed the power of the study to be 57%. A total of 402 knees of 201 participants were evaluated. There were 93 men and 108 women, and the mean age of the participants was 39.23 ± 8.58 (16–50) years. The mean height, weight and body mass index (BMI) of men were 178.40 ± 7.42 cm, 86.01 ± 12.73 kg and 27.02 ± 3.61 kg/m^2^, while they were 164.19 ± 8.35 cm, 76.59 ± 11.97 kg and 28.47 ± 4.49 kg/m^2^ in women. HKA, FMA, TMA, aHKA and JLO in men and women, and in total, are shown in Table 1. HKA (*p* = 0.011), FMA (*p* = 0.017), TMA (*p* < 0.001) and aHKA (*p* < 0.001) were all significantly lower in men than in women, while JLO did not significantly differ between them (*p* = 0.352). ICC of interobserver reliability is detailed in Table 2.

**Table 1:** HKA, FMA, TMA, aHKA and JLO in male, female and total participants.

Angle	Sex						*p* value
	Male		Female		Total		
	Mean ± SD	Range	Mean ± SD	Range	Mean ± SD	Range	
HKA	175.69 ± 2.99	167.00–180.00	176.33 ± 3.00	166.00–180.00	176.03 ± 3.01	166.00–180.00	0.011
FMA	91.69 ± 2.61	83.00–99.00	92.28 ± 2.88	78.00–99.00	92.01 ± 2.77	78.00–99.00	0.017
TMA	86.02 ± 2.60	80.34–92.00	86.94 ± 2.76	77.11–95.45	86.52 ± 2.73	77.11–95.45	<0.001
aHKA	−2.28 ± 3.65	−11.27 to 7.00	−0.79 ± 4.32	−16.49 to 10.13	−1.48 ± 4.09	−16.49 to 10.13	<0.001
JLO	174.33 ± 3.72	163.52–185.83	174.67 ± 3.57	164.51–187.17	174.52 ± 3.64	163.52–187.17	0.352

Abbreviations: aHKA, arithmetic hip–knee–ankle angle; FMA, femoral mechanical axis angle; HKA, hip–knee–ankle angle; JLO, joint line obliquity angle; TMA, tibial mechanical axis angle.

**Table 2:** The table demonstrates the interclass and intraclass correlation coefficients.

Angle	Interclass correlation coefficient	CI	Intraclass correlation coefficient	CI
HKA	0.983	0.968–0.992	0.985	0.964–0.993
FMA	0.960	0.922–0.981	0.930	0.849–0.969
TMA	0.950	0.903–0.976	0.990	0.976–0.995

Abbreviations: CI, confidence interval; FMA, femoral mechanical axis angle; HKA, hip‐knee‐ankle angle; TMA, tibial mechanical axis angle.

### Functional (knee) phenotypes

A total of 75 phenotypes were found in the participants of this study. Table 3 shows the most common phenotypes in overall, men and women. The overall most common phenotypes were VAR_HKA_3°, NEU_FMA_0° and NEU_TMA_0° (Table 4). The distribution of the 10 most common phenotypes was not significantly different in men and women (*p* = 0.201). The phonotypes VAR_HKA_3°, NEU_FMA_0° and NEU_TMA_0° were the most common in women, while the phenotypes NEU_HKA_0°, NEU_FMA_0° and NEU_TMA_0° were the most common in men. Also, a trend of neutral or varus HKA was observed in the 10 most common phenotypes.

**Table 3:** The frequency of the 10 most common phenotypes.

Functional phenotype	Frequency (%)		
	Male	Female	Total
HKAvar3.FMAneu0.TMAneu0	15 (8.1%)	22 (10.2%)	37 (9.2%)
HKAneu0.FMAneu0.TMAneu0	12 (7.0%)	14 (6.5%)	27 (6.7%)
HKAvar3.FMAvar3.TMAneu0	12 (6.5%)	12 (5.6%)	24 (6.0%)
HKAneu0.FMAneu0.TMAval3	6 (3.2%)	14 (6.5%)	20 (5.0%)
HKAvar3.FMAneu0.TMAval3	7 (3.8%)	13 (6.0%)	20 (5.0%)
HKAvar6.FMAvar3.TMAvar3	10 (5.4%)	9 (4.2%)	19 (4.7%)
HKAneu0.FMAval3.TMAneu0	7 (3.8%)	10 (4.6%)	17 (4.2%)
HKAvar3.FMAneu0.TMAvar3	8 (4.3%)	9 (4.2%)	17 (4.2%)
HKAvar6.FMAvar3.TMAneu0	12 (6.5%)	3 (1.4%)	15 (3.7%)
HKAvar3.FMAval3.TMAvar3	9 (4.8%)	6 (2.8%)	15 (3.7%)

**Table 4:** Absolute and relative phenotypes in the participants.

		FMA							
		Varus > 6°	Varus 6°	Varus 3°	Neutral 0°	Valgus 3°	Valgus 6°	Valgus > 6°	Total
TMA	Varus > 6°	0 (0.0%)	0 (0.0%)	1 (0.2%)	0 (0.0%)	0 (0.0%)	0 (0.0%)	0 (0.0%)	1 (0.2%)
	Varus 6°	2 (0.5%)	2 (0.5%)	12 (3.0%)	14 (3.5%)	3 (0.7%)	1 (0.2%)	0 (0.0%)	34 (8.5%)
	Varus 3°	1 (0.2%)	11 (2.7%)	34 (8.5%)	40 (10.0%)	19 (4.7%)	1 (0.2%)	0 (0.0%)	106 (26.4%)
	Neutral 0°	1 (0.2%)	15 (3.7%)	47 (11.7%)	70 (17.4%)	26 (6.5%)	3 (0.7%)	0 (0.0%)	162 (40.3%)
	Valgus 3°	3 (0.7%)	14 (3.5%)	18 (4.5%)	41 (10.2%)	18 (4.5%)	1 (0.2%)	0 (0.0%)	85 (21.1%)
	Valgus 6°	0 (0.0%)	2 (0.5%)	4 (1.0%)	4 (1.0%)	3 (0.7%)	0 (0.0%)	0 (0.0%)	13 (3.2%)
	Valgus > 6°	0 (0.0%)	0 (0.0%)	0 (0.0%)	1 (0.2%)	0 (0.0%)	0 (0.0%)	0 (0.0%)	1 (0.2%)
	Total	7 (1.7%)	34 (8.5%)	116 (28.9%)	170 (42.3%)	69 (17.2%)	6 (1.5%)	0 (0.0%)	402 (100.0%)

Abbreviations: FMA, femoral mechanical axis angle; TMA, tibial mechanical axis.

### CPAK phenotypes

**Figure 1:**
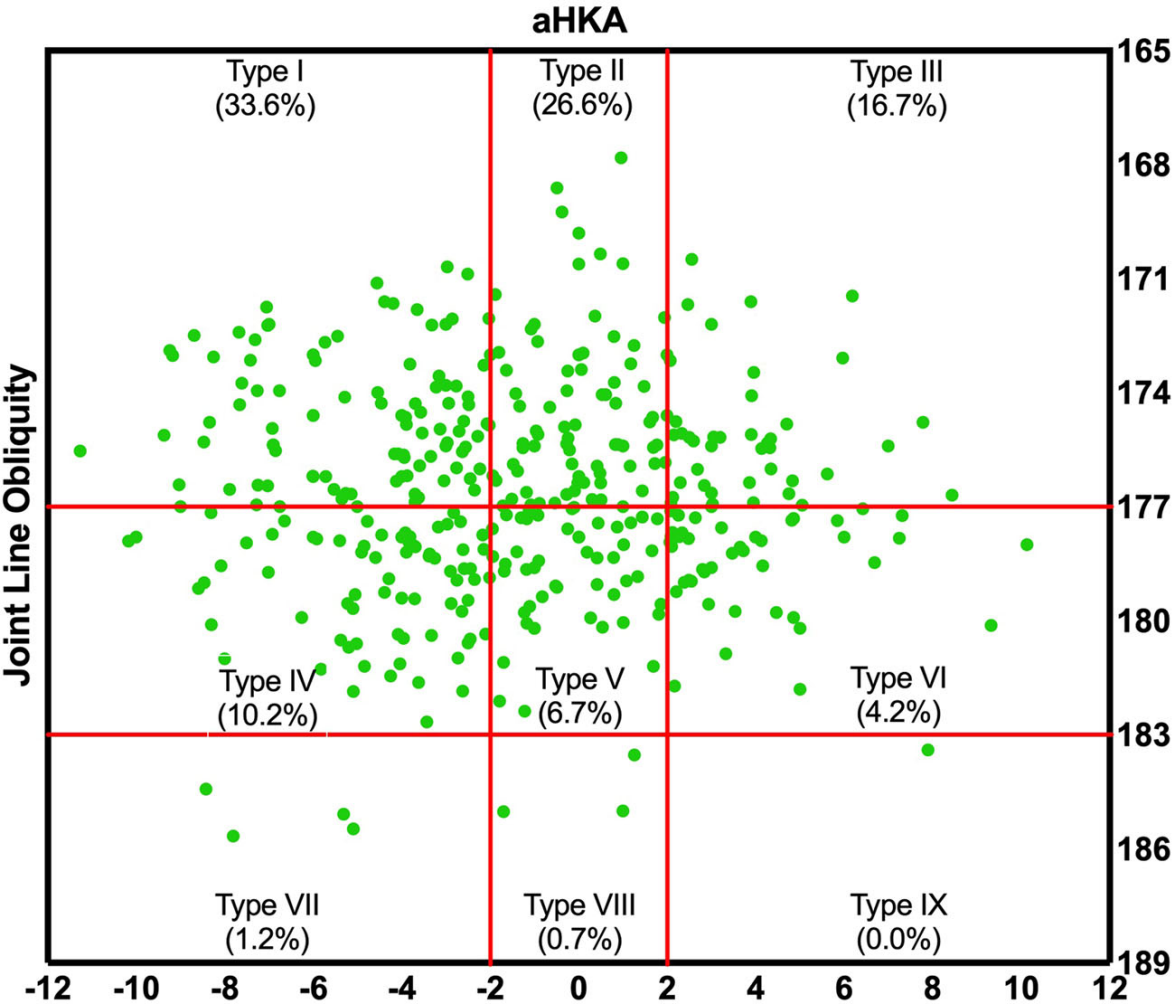
The scatter plot of aHKA and JLO in the healthy population. aHKA, arthritic hip–knee–ankle; JLO, joint line obliquity.

Figure 1 shows the prevalence of the phenotypes in a plot with aHKA against JLO. Most participants in our study showed an apex distal joint line (76.9%), and almost 44% of them had varus alignment (Table 5). The CPAK types were significantly different between men and women (*p* = 0.015) (Table 6). While CPAK types I and II were the most common types in both women and men, women tend to show type III (apex distal JLO and valgus aHKA) more frequently (10.2% in men and 22.2% in women, *p* = 0.001). We did not observe a relationship between BMI and CPAK phenotypes (*p* = 0.514).

**Table 5:** CPAK classification of the knees.

		aHKA			
		Varus	Neutral	Valgus	Total
JLO	Distal	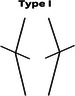 135(33.6%)	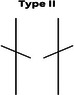 107 (26.6%)	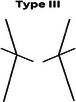 67 (16.7%)	309 (76.9%)
	Neutral	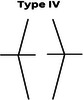 41 (10.2%)	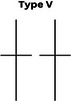 27 (6.7%)	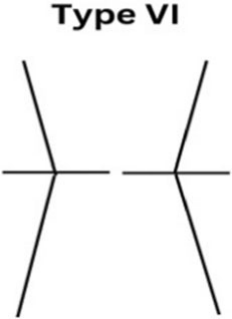 17 (4.2%)	85 (21.1%)
	Proximal	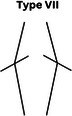 5 (1.2%)	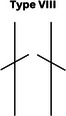 3 (0.7%)	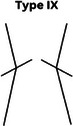 0 (0.0%)	8 (2.0%)
	Total	181 (45.0%)	137 (34.1%)	84 (20.9%)	402 (100.0%)

*Note*: Data are presented as numbers (per cent).

Abbreviations: aHKA, arithmetic hip–knee–ankle angle; JLO, joint line obliquity angle.

**Table 6:** CPAK types in men and women.

Sex	CPAK								
	Type I	Type II	Type III	Type IV	Type V	Type VI	Type VII	Type VIII	Type IX
Male	75 (18.7%)	49 (12.2%)	19 (4.7%)	20 (5.0%)	14 (3.5%)	5 (1.2%)	3 (0.7%)	1 (0.2%)	0 (0.0%)
Female	60 (14.9%)	58 (14.4%)	48 (11.9%)	21 (5.2%)	13 (3.2%)	12 (3.0%)	2 (0.5%)	2 (0.5%)	0 (0.0%)

*Note*: Data are presented as numbers (per cent).

Abbreviation: CPAK, coronal plane alignment knee.

## DISCUSSION

The most important finding of this study was the notable variability in knee alignment phenotypes in a healthy young Iranian population. We also used the CPAK classification to characterize the distribution of these phenotypes. Notably, less than half of the participants exhibited neutral HKA alignment, emphasizing that the neutral alignment targeted by the MA strategy may not represent the natural alignment for a significant portion of the population.

The most common functional knee phenotype observed in our study was VAR_HKA_3°, NEU_FMA_0° and NEU_TMA_0°. Interestingly, we observed a mild varus alignment in the mean HKA (176.03°) in our cohort. The mean HKA, FMA and TMA were all significantly lower in men compared to women. The sex‐specific differences observed in knee alignment—such as significantly lower mean HKA, FMA and TMA in men compared to women—underline the importance of considering patient sex during surgical planning and rehabilitation. The CPAK classification showed that most participants had an apex distal joint line, with nearly 40% presenting with varus alignment. The most common CPAK types identified were types I, II and III, in order. The distribution of CPAK types differed between men and women, with women more frequently exhibiting type III (apex distal JLO and valgus aHKA). We believe that the sex difference that was only observed in the CPAK classification can be due to the fact that Hirschmann differentiates phenotypes into smaller groups that cover a shorter range. It is believed that if the sample size was larger, we might have detected a significant difference between men and women in Hirschmann functional phenotypes. Chelli et al. conducted a large study on 12,099 arthritic knees to evaluate gender differences in alignment and found that males demonstrated higher FMA compared to females, emphasizing the potential influence of sex on knee alignment phenotypes and outcomes [2].

Despite improvements in implant design and surgical techniques, a significant proportion of patients (20%–25%) remain dissatisfied with their functional outcomes [8, 22, 28]. One potential reason for this dissatisfaction is that neutral alignment, the goal of MA, may not represent the natural alignment for all patients [3, 8, 12]. Franceschetti et al. reported that conducting MA strategies for varus CPAK types I and IV can result in inferior PROMs, reinforcing the importance of tailoring alignment strategies to individual phenotypes [6]. Indeed, our study reinforces this idea, as less than half of the healthy non‐osteoarthritic knees in our sample exhibited neutral HKA alignment, supporting the notion that KA strategy may provide more balanced outcomes.

Schelker et al. evaluated different alignment strategies and bone cuts in varus and neutral knees. They concluded that KA strategies produced a JLO closer to the native anatomy in all varus knee phenotypes [27]. Similarly, in neutral knee phenotypes, KA also resulted in a more anatomically native JLO compared to other alignment strategies [28]. Graichen et al. explored the capability of a restricted tibia‐first, gap‐balanced patient‐specific alignment (PSA) technique in restoring bony morphology and alignment phenotypes [9]. They found that PSA effectively restored bony phenotypes and JLO in most straight and varus knees. However, valgus and extreme varus knees were typically normalized rather than restored to the native anatomy.

When comparing our results with those of Hirschmann et al., who identified 43 knee phenotypes, we observed a broader range of variability [13]. The difference may be due to the consideration of only 125 possible phenotypes by Hirschmann et al., while we included all phenotypes in the study. They also reported a prevalence of 5% for patients with the phenotype of MA target (NEU_HKA_0, NEU_FMA_3 and NEU_TMA_3), while we had a 12.8% prevalence in our findings. The most common phenotypes in their study were NEU_HKA_0°, NEU_TMA_0° and NEU_FMA_0°. The most common phenotypes in our findings were VAR_HKA_3°, NEU_FMA_0°, NEU_TMA_0° and NEU_HKA_0°, NEU_FMA_0° and NEU_TMA_0°, in order. We also observed 2 phenotypes with 6° of varus and 5 phenotypes with 3° of varus in HKA in the 10 most common phenotypes. Hirschmann et al. reported no phenotypes with 6° of varus HKA in their findings. These findings may be partly due to higher varus degrees in the Middle Eastern population [4, 5, 15, 18, 19].

Moreover, we found a valgus of 3° in TMA in two phenotypes among the most common phenotypes. Hirschmann et al. reported three phenotypes with VAL_TMA_3°, two of which had an accompanying VAL_HKA_3°. They also reported a phenotype with VAL_FMA_3°, which shows that they had a higher rate of patients with valgus.

We found the most frequent CPAK types to be I, II and III. MacDessi et al. reported types II, I and V to be the most common in 500 healthy knees in Belgium [20]. Overall, there seems to be a trend towards the varus alignment in our healthy participants. Steele et al. reported the classification in 1946 knees in the United States and found that the most frequent types were type II, I and III, respectively [30]. Although they used a mixed population of healthy and osteoarthritic participants.

Another study by Mulpur et al. on 500 healthy and 500 arthritic knees in India showed that types II, I and V were the most frequent in the healthy knees, and types I, IV and II were the most common in the osteoarthritic knees [21]. Gao et al. evaluated the alignment of 214 healthy knees with 477 arthritic knees in the Chinese population [7]. They found that CPAK types I, II and IV were the most frequent in arthritic knees, and CPAK types II, III and I were the most common in healthy knees. Yang et al. evaluated the alignment of 500 healthy knees with 500 arthritic knees in the Korean population [34]. They found that CPAK types I, II and IV were the most frequent in arthritic knees, and CPAK types II, I and III were the most common in healthy knees. Hsu et al. also evaluated the alignment of 214 knees of healthy young Taiwanese adults and found the CPAK types II, I, and III to be the most common. They found no cases of types VII and VIII [16]. In contrast, our findings diverged from the existing literature regarding CPAK distribution, suggesting a potential influence of ethnicity on knee phenotypes. However, further multicenter studies involving diverse populations are needed to substantiate this hypothesis and elucidate potential genetic, environmental or lifestyle factors contributing to these differences. However, a consistent trend across all studies, including ours, is the predominance of knees with an apex distal JLO, while apex proximal knees were rarely observed.

The clinical implications of these findings are significant. Understanding the natural variability in knee alignment phenotypes can enhance preoperative planning, ensuring that surgical interventions are better aligned with a patient's unique anatomy. This individualized approach could improve implant longevity, patient satisfaction and functional outcomes. Furthermore, recognizing the ethnic and sex‐specific variations in knee alignment can inform the development of population‐specific surgical protocols and rehabilitation strategies, potentially reducing postoperative complications and enhancing recovery trajectories.

## LIMITATIONS

Several limitations should be considered. First, this cross‐sectional design does not allow for longitudinal assessment of changes in knee alignment over time, particularly as participants age or develop osteoarthritis. Second, the study sample was drawn from a single centre in Iran, which may limit the generalizability of our findings. However, the centre serves as a major tertiary referral hospital, receiving patients from across the country, which enhances the diversity of the sample to some extent. Additionally, while we employed well‐established radiographic methods for evaluating alignment, variability in positioning during imaging could have influenced the measurements. Despite efforts to standardize radiographic techniques and ensure interobserver and intraobserver reliability, the potential for minor measurement inaccuracies remains. Finally, while our sample size of 402 knees provided sufficient power for most analyses, it may not be large enough to capture rarer knee phenotypes, especially in subgroups such as those with extreme varus or valgus alignment.

## CONCLUSION

In conclusion, our study reveals that knee alignment in the healthy Iranian population tends to exhibit neutral or mild varus characteristics, with significant differences between men and women. These findings suggest that ethnicity may play a role in the natural alignment of the knee, which has important implications for surgical planning and outcomes.

## AUTHOR CONTRIBUTIONS

M.R and S.M.J.M contributed to the study conception and design, analyzed data and edited the manuscript. M.S. contributed to the study design and wrote the first draft of the manuscript. A.M. and M.A.F. contributed to the study design and data collection and drew figures. All authors commented on previous versions of the manuscript and revised it. All authors read and approved the final manuscript.

## CONFLICT OF INTEREST STATEMENT

The authors declare no conflicts of interest.

## ETHICS STATEMENT

The study was reviewed and approved by the Institutional Review Board of Tehran University of Medical Sciences. Approval ID: IR.TUMS.IKHC.REC.1403.002. All participants provided informed consent, including consent for the publication of data and photographs.

## Data Availability

The data that support the findings of this study are available from the corresponding author, Seyed Mohammad Javad Mortazavi, upon reasonable request.
